# Distribution of lymph node metastases in esophageal carcinoma [TIGER study]: study protocol of a multinational observational study

**DOI:** 10.1186/s12885-019-5761-7

**Published:** 2019-07-04

**Authors:** Eliza R. C. Hagens, Mark I. van Berge Henegouwen, Johanna W. van Sandick, Miguel A. Cuesta, Donald L. van der Peet, Joos Heisterkamp, Grard A. P. Nieuwenhuijzen, Camiel Rosman, Joris J. G. Scheepers, Meindert N. Sosef, Richard van Hillegersberg, Sjoerd M. Lagarde, Magnus Nilsson, Jari Räsänen, Philippe Nafteux, Piet Pattyn, Arnulf H. Hölscher, Wolfgang Schröder, Paul M. Schneider, Christophe Mariette, Carlo Castoro, Luigi Bonavina, Riccardo Rosati, Giovanni de Manzoni, Sandro Mattioli, Josep Roig Garcia, Manuel Pera, Michael Griffin, Paul Wilkerson, M. Asif Chaudry, Bruno Sgromo, Olga Tucker, Edward Cheong, Krishna Moorthy, Thomas N. Walsh, John Reynolds, Yuji Tachimori, Haruhiro Inoue, Hisahiro Matsubara, Shin-ichi Kosugi, Haiquan Chen, Simon Y. K. Law, C. S. Pramesh, Shailesh P. Puntambekar, Sudish Murthy, Philip Linden, Wayne L. Hofstetter, Madhan K. Kuppusamy, K. Robert Shen, Gail E. Darling, Flávio D. Sabino, Peter P. Grimminger, Sybren L. Meijer, Jacques J. G. H. M. Bergman, Maarten C. C. M. Hulshof, Hanneke W. M. van Laarhoven, Banafsche Mearadji, Roel J. Bennink, Jouke T. Annema, Marcel G. W. Dijkgraaf, Suzanne S. Gisbertz

**Affiliations:** 1 Department of Surgery, Cancer Center Amsterdam, Amsterdam UMC, University of Amsterdam, Meibergdreef 9, Amsterdam, Netherlands; 2 Netherlands Cancer Institute, Amsterdam, The Netherlands; 3 Department of Surgery, Amsterdam UMC, Vrije Universiteit Amsterdam, De Boelelaan, 1117 Amsterdam, Netherlands; 4 Elisabeth-TweeSteden Ziekenhuis, Tilburg, The Netherlands; 5 Catharina Ziekenhuis, Eindhoven, The Netherlands; 6 Radboud universitair medisch centrum, Nijmegen, The Netherlands; 7 Reinier de Graaf Gasthuis, Delft, The Netherlands; 8 Atrium Medical Center, Heerlen, The Netherlands; 9 Universitair Medisch Centrum Utrecht, Utrecht, The Netherlands; 10 Erasmus MC, Rotterdam, The Netherlands; 11 Karolinska Institutet, Stockholm, Sweden; 12 Hospital District of Helsinki and Uusimaa, Helsinki, Finland; 13 Universitair Ziekenhuis Leuven, Leuven, Belgium; 14 Universitair Ziekenhuis, Ghent, Belgium; 15 Agaplesion Markus Krankenhuis, Frankfurt am Main, Germany; 16 Uniklinik Köln, Cologne, Germany; 17 Triemli Medical Center and Hirslanden Medical Center, Zürich, Switzerland; 18 University Hospital C. Huriez Place de Verdun, Lille, France; 19 Humanitas University Hospital, Milan, Italy; 20 Istituto di Ricovero e Cura a Carattere Scientifico, Policlinico San Donato, University of Milano, Milan, Italy; 21 Ospedale San Raffaelo, Milan, Italy; 22 University of Verona, Verona, Italy; 23 Universita di Bologna, Bologna, Italy; 24 l’Hospital Josep Trueta, Girona, Spain; 25 Hospital Universitario del Mar, Barcelona, Spain; 26 Royal Victoria Infirmary, New Castle upon Tyne Hospitals, New Castle, UK; 27 University Hospitals Bristol, Bristol, UK; 28 The Royal Marsden, London, UK; 29 Oxford University Hospitals, Oxford, UK; 30 Heart of England Foundation Trust, Birmingham, UK; 31 Norfolk and Norwich University Hospital, Norwich, UK; 32 Imperial College, London, UK; 33 Connolly Hospital Blanchardstown, Dublin, Ireland; 34 Trinity College, Dublin, Ireland; 35 National Cancer Center Hospital, Tokyo, Japan; 36 Showa University, Northern Yokohama Hospital, Yokohama, Japan; 37 Chiba University, Graduate School of Medicine, Chiba, Japan; 38 Uonuma Institute of Community Medicine, Niigata University Medical and Dental Hospital, Minami-Uonuma, Japan; 39 Fudan University Shanghai Cancer Center, Shanghai, China; 40 University of Hong Kong, Pok Fu Lam, Hong Kong; 41 Tata Memorial Centre, Mumbai, India; 42 Galaxy Care Laparoscopy Institute, Pune, Maharashtra India; 43 Cleveland Clinics, Cleveland, OH USA; 44 University Hospitals, Cleveland, USA; 45 MD Anderson, Houston, USA; 46 Virginia Mason Medical Center, Seattle, USA; 47 Mayo Clinic, Rochester, USA; 48 University of Toronto, Toronto, Canada; 49 Instituto Nacional de Câncer, Rio de Janeiro, Brazil; 50 University Medical Center of the Johannes Gutenberg University, Mainz, Germany; 51 Department of Gastro-Intestinal Surgery, Amsterdam UMC, location AMC, PO Box 22660, 1100 DD Amsterdam, The Netherlands

**Keywords:** Esophageal cancer, Lymph node metastases, Lymphadenectomy, Esophagectomy

## Abstract

**Background:**

An important parameter for survival in patients with esophageal carcinoma is lymph node status. The distribution of lymph node metastases depends on tumor characteristics such as tumor location, histology, invasion depth, and on neoadjuvant treatment. The exact distribution is unknown. Neoadjuvant treatment and surgical strategy depends on the distribution pattern of nodal metastases but consensus on the extent of lymphadenectomy has not been reached. The aim of this study is to determine the distribution of lymph node metastases in patients with resectable esophageal or gastro-esophageal junction carcinoma in whom a transthoracic esophagectomy with a 2- or 3-field lymphadenectomy is performed. This can be the foundation for a uniform worldwide staging system and establishment of the optimal surgical strategy for esophageal cancer patients.

**Methods:**

The TIGER study is an international observational cohort study with 50 participating centers. Patients with a resectable esophageal or gastro-esophageal junction carcinoma in whom a transthoracic esophagectomy with a 2- or 3-field lymphadenectomy is performed in participating centers will be included. All lymph node stations will be excised and separately individually analyzed by pathological examination. The aim is to include 5000 patients. The primary endpoint is the distribution of lymph node metastases in esophageal and esophago-gastric junction carcinoma specimens following transthoracic esophagectomy with at least 2-field lymphadenectomy in relation to tumor histology, tumor location, invasion depth, number of lymph nodes and lymph node metastases, pre-operative diagnostics, neo-adjuvant therapy and (disease free) survival.

**Discussion:**

The TIGER study will provide a roadmap of the location of lymph node metastases in relation to tumor histology, tumor location, invasion depth, number of lymph nodes and lymph node metastases, pre-operative diagnostics, neo-adjuvant therapy and survival. Patient-tailored treatment can be developed based on these results, such as the optimal radiation field and extent of lymphadenectomy based on the primary tumor characteristics.

**Trial registration:**

NCT03222895, date of registration: July 19th, 2017.

## Background

Survival rates following an esophageal resection for esophageal carcinoma vary from a median of 25 to 74 months [1, 2]. An important parameter for survival is lymph node status [3–12]. The distribution of lymph node metastases depends on tumor characteristics such as tumor location, histology, invasion depth, and on neoadjuvant treatment [13–20]. However, the precise distribution pattern is unknown.

In the Netherlands, among other countries, curative treatment for patients with esophageal carcinoma consists of neoadjuvant chemoradiation followed by surgery [21, 22]. The radiation field during neoadjuvant treatment and the lymphadenectomy during surgery depend on the location of lymph node metastases but no consensus has been reached on the extent of the lymphadenectomy [23]. If a distribution pattern of lymph node metastases of esophageal carcinoma can be identified, the optimal neoadjuvant and surgical treatment can be determined.

The administration of neoadjuvant therapy itself can also influence the distribution of lymph node metastases [13]. Neoadjuvant chemoradiation may be able to sterilize metastatic lymph nodes. Lymph nodes inside the radiation field are affected by both radiotherapy and concurrent chemotherapy, whereas lymph nodes outside the radiation field are affected by chemotherapy only. Nevertheless, recent studies show that also after neoadjuvant therapy, the extend of lymphadenectomy is directly related to survival [24]. Therefore, not only the metastatic behavior of untreated esophageal carcinoma is an important factor, also the pattern after neoadjuvant therapy can provide valuable information for the optimal extend of lymphadenectomy after chemo (radio)therapy.

Especially for adenocarcinoma the distribution of lymph node metastases has not yet been described in large series that report on a complete 2- or 3-field lymphadenectomy [13, 14]. Also, different classification systems for lymph node stations are used in current literature, leading to incomparable studies. Besides the limited and heterogeneous evidence, also the significant morbidity involved in esophageal surgery makes the treatment choices demanding considering that the removal of more lymph nodes may lead to a more invasive procedure, possibly increasing the risk for postoperative morbidity.

A large observational study could identify lymph node stations that should be resected in relation to tumor characteristics and may clarify if the same surgical strategy is justified in patients with and without neoadjuvant therapy. Furthermore, the prognostic value of different lymph node stations can be established. We thus propose a multicenter prospective study to determine the distribution of lymph node metastases in patients with resectable esophageal or gastro-esophageal junction carcinoma in whom a transthoracic esophagectomy with a 2- or 3-field lymphadenectomy is performed. This can be the foundation for a uniform worldwide staging system and establishment of the optimal surgical strategy for esophageal cancer patients.

## Methods

### Objective

The aim of the TIGER study is to evaluate the distribution of lymph node metastases in esophageal carcinoma specimens following transthoracic esophagectomy with a 2- or 3- field lymphadenectomy.

### Study design and setting

TIGER is an international observational cohort study. The duration of the study will approximately be 7 years (2 years inclusion, 5 years follow-up). There are currently 50 participating centers distributed over 18 countries. Centers are located in The Netherlands (10), Sweden (1), Finland (1), Belgium (2), Germany (3), Swiss (1), France (1), Italy (5), Spain (2), United Kingdom (7), Ireland (2), Japan (4), Hong Kong (1), China (1), India (2), United States (5), Canada (1) and Brazil (1). Data from each participating hospital will be collected at the TIGER website (www.tigerstudy.net). Data collection forms can be assessed after login on the website. Each hospital has access to their own dataset.

### Study population

All patients with a resectable (cT1-4a, N0–3, M0) esophageal or gastro-esophageal junction carcinoma.

#### Inclusion criteria

In order to be eligible to participate in this study, a subject must meet all of the following criteria:

- Primary squamous cell or adenocarcinoma of the esophagus or esophago-gastric junction
- Surgically resectable tumor (cT1-4a, N0–3, M0)
- Adequate physical condition to undergo transthoracic surgery (ASA 1–3)
- Transthoracic esophagectomy, either open or minimal invasive

#### Exclusion criteria

A potential subject who meets any of the following criteria will be excluded from participation in this study:

- Previous thoracic or abdominal (upper GI) surgery disturbing lymph drainage of the esophagus and stomach
- Patients with in situ carcinoma or high-grade dysplasia

### Sample size

The aim is to include 5000 patients. This number suffices for descriptive purposes and clustering of metastases diffusion profiles into meaningful subgroups within predefined strata (patients with adenocarcinoma or squamous cell carcinoma, with and without neoadjuvant therapy, different tumor heights and invasion depths, and following a 2- or 3-field lymphadenectomy). In 2012, the incidence of esophageal cancer was 456.000 new cases worldwide [25]. Only a small percentage of patients with esophageal cancer present with curable disease at time of diagnosis. The aim is to include all 5000 patients with resectable disease in the 50 participating centers in a 2-year time period.

### Primary endpoint

The distribution of lymph node metastases in esophageal and esophago-gastric junction carcinoma specimens following transthoracic esophagectomy with at least 2-field lymphadenectomy in relation to tumor histology, tumor location, invasion depth and neoadjuvant therapy.

### Secondary endpoints

- Accuracy of preoperative diagnostics (EUS and PET-CT) and added value of EBUS to existing staging with EUS/PET-CT
- Prognostic value of different lymph node stations
- Postoperative morbidity (anastomotic leakage, chyle leakage, pneumonia, recurrent nerve injury and arrhythmia)
- 30-days/in-hospital and 90-days mortality
- 3- and 5-year overall and disease-free survival
- Distribution pattern of recurrence or metastases
  - In-field- or out-field nodal recurrence in case of neo-adjuvant chemoradiation
- Number of harvested lymph nodes in patients who are treated with and without neo-adjuvant chemo (radio)therapy
- Analysis of the phenomenon skip nodal metastases
- Ratio of nodal metastases inside and outside the radiation field

### Treatment of subjects

#### Staging

Preoperative staging will be performed according to national guidelines. This therefore, may differ per country. Usually, patients are staged with an endoscopy with biopsies, an endoscopic ultrasound, a PET-CT-scan of neck, thorax and abdomen and an ultrasound of the neck. An EBUS-TBNA will be performed if indicated. All patients are discussed in a multidisciplinary team (MDT).

A suspected lymph node is defined as a node larger than > 9 mm short axis or a node 5–9 mm short axis that is round, inhomogeneous and has an irregular border (2 out of 3). In case of PET-CT-scan, pathological nodes are those with FDG uptake, a short axis > 10 mm or those with a short axis between 5 and 9 mm that present with sharp borders.

#### Neoadjuvant therapy

In case of potentially curative disease (cT1-4a, N0–3, M0) patients may be treated with neoadjuvant therapy, this however, may differ per country and usually consists of chemotherapy or chemoradiotherapy.

#### Radiation fields

One coronal, 1 sagittal and 3 axial CT-images of the radiation field is documented in the medical file, to assess lymph nodes stations incorporated in the radiation field. An anatomical description of the radiation field will be given according to highest and lowest radiated lymph node station. An in-field lymph node is defined as a lymph node within the clinical target volume. Clinical target volume fields as defined gross tumor volume plus a margin for sub-clinical disease spread.

#### Restaging

After completion of neoadjuvant therapy patients will generally be restaged with a (PET-)CT scan to exclude distant metastases before patients are scheduled for surgery. In addition, the locoregional lymph nodes will be evaluated. The (PET-)CT will usually be performed 2–4 weeks after completion of neoadjuvant therapy.

#### Surgery

If no metastases are detected, patients will be operated 5–12 weeks after completion of neoadjuvant therapy. This may differ per country and may depend on the type of neoadjuvant therapy. If no neoadjuvant therapy is administered patients are directly scheduled for surgery.

An esophageal resection with a 2- or 3-field lymphadenectomy can be performed via a thoracolaparoscopy, a thoracolaparotomy or a hybrid procedure and a cervical incision as indicated. A gastric tube or colonic interposition can be used for reconstruction. Lymph node stations may be dissected ex vivo (after enbloc resection with the specimen), in vivo, or partially ex and in vivo, and will be separately sent for pathological examination. Lymph node stations in close proximity to the tumor are preferably marked with sutures or beads to prevent damage to the specimen and circumferential resection margin.

#### Lymph node classification systems

**Fig. 1:**
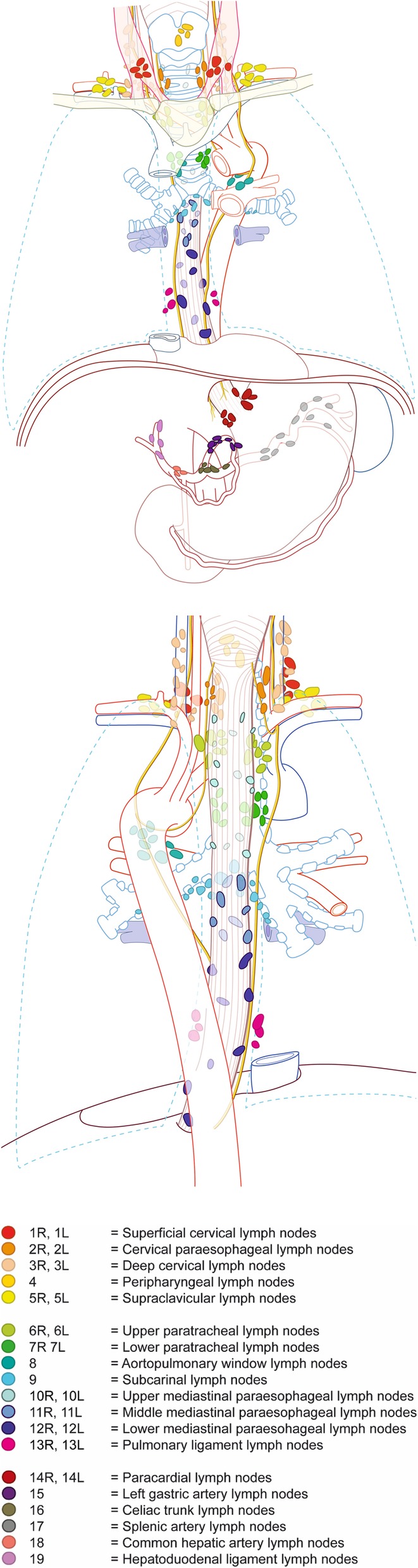
Classification of lymph node stations.

Different classification systems are used in different countries to classify lymph node stations around the esophagus: the AJCC 8th edition esophageal cancer staging and the JES 11th edition esophageal cancer staging. In Fig. 1 and Table 1 these classifications have been combined for the purpose of this study.

**Table 1:** Classification of lymph node stations

Cervical lymph node stations (based on the JES 11th edition esophageal cancer staging)	
1. Superficial cervical lymph nodes	
• Lymph nodes located along the external jugular veins and anterior jugular veins beneath the superficial cervical fascia. • Lymph nodes located around the submandibular glands and parotid glands, and anterior to the mylohyoid muscle. • Lymph nodes located in the pretracheal fatty tissue, extending from the hyoid bone superiorly, to the left brachiocephalic vein inferiorly, including the prethyroidal lymph nodes and the prelaryngeal lymph nodes. • Lymph nodes located along the accessory nerve(s), and anterior to the trapezius muscle.	
2. Cervical paraesophageal lymph nodes	
• Lymph nodes located around the cervical esophagus, including lymph nodes located along the recurrent laryngeal nerve and the cervical paratracheal lymph nodes. The lateral boundary is the medial border of the carotid sheath.	
3. Deep cervical lymph nodes	
• Lymph nodes located around the internal jugular vein and the common carotid artery. • Lymph nodes located from the caudal border of the digastric muscle superior to the carotid artery bifurcation. • Lymph nodes located from the carotid artery bifurcation superiorly to the lower border of the cricoid cartilage inferiorly.	
4. Peripharyngeal lymph nodes	
• Lymph nodes located medial to the carotid sheath, extending from the caudal border of the digastric muscle superiorly to the lower border of the cricoid cartilage inferiorly. Postpharyngeal and parapharyngeal lymph nodes are included.	
5. Supraclavicular lymph nodes	
• Lymph nodes located in the supraclavicular fossa, extending from the lower border of the cricoid cartilage superiorly, to the clavicle inferiorly, including the lower internal deep cervical lymph nodes. The medial boundary is the medial border of the carotid sheath.	
Thoracic lymph node stations (based on the JES 11th edition esophageal cancer staging and the AJCC 8th edition esophageal cancer staging)	
6. Upper paratracheal lymph nodes (right / left)	
• Right: Lymph nodes located around the upper thoracic esophagus posterior to the right vagal nerve. Lymph nodes located along the anterior and lateral wall of the thoracic trachea until the level of the right vagal nerve. Lymph nodes located along the right recurrent laryngeal nerve in the mediastinum. The superior boundary is drawn from the cephalic border of the right subclavian artery to the suprasternal notch. • Left: Lymph nodes located around the upper thoracic esophagus. Lymph nodes located along the anterior and lateral wall of the thoracic trachea until the upper margin of the aortic arch. Lymph nodes located along the left recurrent laryngeal nerve in the mediastinum. The superior boundary is drawn from the cephalic border of the left subclavian artery to the suprasternal notch.	
7. Lower paratracheal lymph nodes (right / left)	
• Right: Lymph nodes located in the tracheobronchial angle and located along the anterior and lateral wall of the thoracic trachea. The superior boundary is the vagal nerve, the ventral boundary the superior caval vein. • Left: Lymph nodes located in the tracheobronchial angle and located along the anterior and lateral wall of the thoracic trachea. Lymph nodes located along the azygos vein arch and the right bronchial artery are included. Lymph nodes along the proximal part of the left recurrent laryngeal nerve along the aortic arch are also included. The superior boundary is the inferior wall of the aortic arch, and the lymph nodes are located in the area surrounded by the medial wall of the aortic arch.	
8. Aortopulmonary window lymph nodes	
• Subaortic and para-aortic nodes lateral to the ligamentum arteriosum. Superior boundary is the lower margin of the aortic arch. Ventral boundary is the pulmonary artery, distal boundary the left main bronchus.	
9. Subcarinal lymph nodes	
• Lymph nodes located caudal to the carina of the trachea. The lateral boundaries are the extended line of both lateral margins of the trachea.	
10. Upper mediastinal paraesophageal lymph nodes • Dissection of the lymph nodes located around the upper thoracic esophagus. From the thoracic aperture until the trachea bifurcation.	
11. Middle mediastinal paraesophageal lymph nodes	
• Lymph nodes located around the middle thoracic esophagus. From the trachea bifurcation to the caudal margin of the inferior pulmonary vein.	
12. Lower mediastinal paraesophageal lymph nodes	
• Lymph nodes located around the lower thoracic esophagus. From the caudal margin of the inferior pulmonary vein to the esophagogastric junction	
13. Pulmonary ligament lymph nodes (right / left)	
• Right: Dissection of the lymph nodes within the right inferior pulmonary ligament. • Left: Dissection of the lymph nodes within the left inferior pulmonary ligament.	
Abdominal lymph node stations (based on the JES 11th edition esophageal cancer staging ans the AJCC 8^th^edition esophageal cancer staging)	
14. Paracardial lymph nodes (right / left)	
• Right: Lymph nodes located immediately adjacent to the gastroesophageal junction, including those along the first branch of the ascending limb of the left gastric artery. • Left: Lymph nodes located immediately adjacent to the gastroesophageal junction, including those along the esophagocardiac branch of the left subphrenic artery	
15. Left gastric artery lymph nodes	
• Lymph nodes along the course of the left gastric artery. Superior boundary is the caudal border of the first branch of the ascending limb of the left gastric artery. Proximal boundary is the origin of the left gastric artery.	
16. Celiac trunk lymph nodes	
• Lymph nodes located around the celiac trunk. Dorsal boundary is the aorta; ventral boundary is the origin of the left gastric artery.	
17. Splenic artery and splenic hilum lymph nodes	
• Lymph nodes from the origin of the splenic artery along its course alongside the pancreatic tail, including those adjacent to the splenic artery distal to the pancreatic tail, and those on the roots of the short gastric arteries and those along the left gastroepiploic artery proximal to its 1st gastric branch.	
18. Common hepatic artery lymph nodes	
• Lymph nodes from the origin of the common hepatic artery along its course until the division into the gastroduodenal and proper hepatic artery.	
19. Hepatoduodenal ligament lymph nodes	
• Lymph nodes along the proper hepatic artery and along the portal vein in the caudal half between the confluence of the right and left hepatic ducts and the upper border of the pancreas.	

In case of a 3-field lymphadenectomy stations 1–19 will be resected and in case of a 2-field lymphadenectomy stations 6–19 will be resected as usual. If not all lymph node stations are resected, patients can still be included in the study, however this has to be documented accurately. A video appendix and definitions of the individual lymph node stations are displayed and available for review on the TIGER study website.

#### Pathology

The esophageal resection and lymphadenectomy specimens will be processed and analyzed according to national and international guidelines by the department of pathology. In addition to the separately sent lymph node stations, the esophageal surgical resection specimen will be carefully analyzed for retained lymph nodes and structures macroscopically suspicious for lymph nodes will be embedded. All lymph nodes under 5 mm will be totally embedded for microscopic evaluation, larger lymph nodes will be totally embedded in slices of 3–4 mm thick. Microscopically, a circumscript area of lymphoid cells containing a follicular architecture and/or a subcapsular sinus is identified as a lymph node. In the final report the exact localization and number of lymph nodes found will be reported.

Initial microscopic evaluation will be performed by standard H&E staining. In case of suspicion of micro-metastasis (0.2–2.0 mm) or isolated tumor cells in the lymph node, or in case of suspicion of residual tumor cells in patients with extensive response to neoadjuvant therapy, additional keratin stains will be performed. A metastatic lymph node is defined as a lymph node with tumor cells. Lymph nodes containing micro-metastasis or isolated tumor cells are also considered as metastatic lymph nodes but will be also recorded separately. Lymph nodes with regression after chemoradiation with isolated tumor cells will be considered as metastatic lymph nodes, however, these also will be recorded separately, so that the prognostic value of these isolated tumor cells can be determined. The same applies to fibrotic lymph nodes without vital tumor cells. These are so called preneoadjuvant therapy N+ nodes. In case of an adenocarcinoma of the esophagus, Her2-status will be analyzed (immunohistochemistry and SISH).

#### Follow-up

Patients will be followed up until 5 years after the operation. Follow-up will usually be scheduled (but differences between countries exist) every 3 months the first year, every 6 months the second until the fourth year and once yearly until the fifth year. Investigations are performed according to national guidelines.

### Statistical analysis

#### Primary study parameter(s)

Numbers and percentages of resected lymph nodes and lymph node metastases will be given per lymph node station (Fig. 1). Tumor location and invasion depth will be categorized. Patients with adenocarcinoma and squamous cell carcinoma and patients with and without neoadjuvant therapy will be analyzed separately.

#### Secondary study parameter(s)

The sensitivity, specificity, and positive and negative predictive values of EUS and PET-CT will be reported. Perioperative morbidity and mortality will be summarized descriptively. For each patient group (squamous cell versus adenocarcinoma, with and without neoadjuvant therapy), explorative cluster analyses will be performed to identify subgroups of patients with different patterns of lymph nodes metastases. Potentially relevant characteristics at the time of surgery like age, gender, tumor location, tumor invasion depth, tumor differentiation, vaso-invasive growth will be included in the analysis. No restrictions will be applied to the number of clusters in each analysis, but the ratio of the largest cluster size to the smallest cluster size should preferably not exceed the value of 3 and/or the smallest cluster size should be minimally 30 patients. Characteristics introducing patient outliers will be excluded and one should further be able to attribute meaning to the resulting cluster profiles. Clusters that show the phenomenon of skip metastases will be noted. The resulting clusters will be evaluated for the diffusion pattern of future metastases during follow-up (descriptive analysis), the number of future metastases during follow-up (Poisson regression or generalized estimation equation, whichever appropriate), for 3- and 5-year overall and disease-free survival (Kaplan-Meier survival analysis). Multivariate analysis will be performed using the Cox hazard regression method. The univariate analysis, including all baseline parameters, will serve as the basis for the multivariate Cox hazard regression model. Variables showing association (*p* < 0.10) with survival in univariate analysis will be included in the multivariate analysis. Age and sex will be included in all multivariate analyses. Results are presented as hazard ratio with exact 95% confidence interval (95% CI). After 5-years of follow-up the efficacy index will be determined (incidence of metastases to an area (%) × 5-year overall survival rate (%)). A log-rank test, Mann-Whitney U test, or χ^2^-test will be used as indicated to compare groups. A value of *p* < 0.05 will be considered statistically significant.

## Discussion

The TIGER study is an international observational cohort study, with participation of worldwide renowned esophageal cancer centers, that will investigate the distribution of lymph node metastases is esophageal cancer. This global study group will be a unique international collaboration that will determine the pattern of lymph node metastases in both squamous cell and adenocarcinoma, since, especially in adenocarcinoma, this has not been investigated in multicenter prospective series before [11, 12]. The results of the TIGER study will provide a roadmap of the location of lymph node metastases in relation to tumor histology, tumor location, tumor invasion depth, number of lymph nodes and lymph node metastases, pre-operative diagnostics, neoadjuvant therapy and survival. In addition, it will be investigated whether neoadjuvant chemoradiotherapy and chemotherapy influence the presence and location of lymph node metastases, as some recent reports suggest that lymph nodes can become sterile after neoadjuvant treatment and only fibrosis is found in these lymph nodes [22]. The prognostic value of positive lymph nodes, micrometastases, isolated tumor cells and fibrosis will be investigated. Patient-tailored treatment can be developed on the basis of the TIGER study results, such as the optimal radiation field and the extent of the lymphadenectomy and, additionally, it may aid in the development of a global uniform classification system.

## Trial status

Protocol version 6, date: 08-02-2019

Start recruitment: 03-03-2019

Approximate date recruitment completion: 03-03-2021

## Data Availability

The datasets generated and/or analyzed during the current study will be available from the corresponding author on reasonable request.

## Abbreviations

AJCC: American Joint Committee on Cancer
EBUS-TBNA: Endobronchial ultrasound-transbronchial needle aspiration
EUS: Endoscopic ultrasound
H&E: Hematoxylin and eosin stain
IRB: Investigational Research Board
JES: Japan Esophageal Society
MDT: Multidisciplinary team
PET-CT: Positron emission tomography-computed tomography
